# Nm23‐H1 inhibits lung cancer bone‐specific metastasis by upregulating miR‐660‐5p targeted SMARCA5

**DOI:** 10.1111/1759-7714.13308

**Published:** 2020-02-05

**Authors:** Cheng Ai, Guangzhi Ma, Yunfu Deng, Qiangqiang Zheng, Yingcai Gen, Wen Li, Yang Li, Lingling Zu, Qinghua Zhou

**Affiliations:** ^1^ Lung Cancer Center West China Hospital, Sichuan University Chengdu China; ^2^ Department of Cardiothoracic Surgery Panzhihua Central Hospital of Sichuan Panzhihua China; ^3^ Tianjin Key Laboratory of Lung Cancer Metastasis and Tumor Microenvironment, Tianjin Lung Cancer Institute Tianjin Medical University General Hospital Tianjin China

**Keywords:** Lung cancer, miR‐660‐5p, nm23‐H1, RANKL, SMARCA5

## Abstract

**Background:**

Nm23‐H1 gene has been found to be an inhibitor of tumor metastasis in lung cancer. MicroRNAs (miRNAs) play key roles in tumor metastasis through multiple signaling pathways. This study explored whether the nm23‐H1 gene could inhibit invasion and metastasis of lung cancer cells by regulating miRNA‐660‐5p targets.

**Methods:**

Quantitative real‐time PCR (qRT‐PCR) and western blots were used to measure the expression of nm23‐H1 and miR‐660‐5p of various human lung cancer cell lines. Cell counting kit‐8 (CCK‐8), wound‐healing and transwell assay were carried out to assess cell proliferation, migration and invasion of each cell line. Xenograft were applied to determine in vivo effects of miR‐660‐5p among nude mice. Luciferase assay and western blot were performed to determine the target gene of miR‐660‐5p.

**Results:**

We found that high expression of nm23‐H1 correlated with decreased miRNA‐660‐5p expression. Inhibiting miR‐660‐5p suppressed lung cancer cells progression significantly in vitro, whereas overexpression of miR‐660‐5p facilitated tumor growth and bone metastasis in vivo. In addition, as the potential target gene of miR‐660‐5p, SMARCA5 overexpression in vitro suppressed tumor progression and osteolytic metastasis associated RANKL signaling, which is congruent with the effect of nm23‐H1 on the lung cancer cells.

**Conclusion:**

Nm23‐H1 inhibits tumor progression and bone‐specific metastasis of lung cancer by regulating miR‐660‐5p/SMARCA5/RANKL axis, which indicates the related genes may serve as potential targets for the treatment of human lung cancer.

**Key points:**

Significant findings of the study

High expression of nm23‐H1 correlated with decreased miRNA‐660‐5p expression. Further, downregulation of miR‐660‐5p significantly suppressed the tumor progression and bone‐specific metastasis of lung cancer cells.

What this study adds

This is the first study to show an inverse association between nm23‐H1 and miR‐660‐5p, and confirm that nm23‐H1 inhibits tumor progression and bone‐specific metastasis of lung cancer by regulating miR‐660‐5p/SMARCA5/RANKL axis.

## Introduction

Lung cancer is one of the most common malignant tumors globally and is a threat to human health and quality of life.^1^ Among all human cancers, the disease has the highest morbidity and mortality worldwide.^2^ Despite recent progress in multimodal management, lung cancer prognosis remains poor, primarily because of its aggressive metastasis to various organs.^3^ Cancer cells typically spread to the lymph nodes, bone, brain, and liver. The skeleton is a frequent target of lung cancer metastasis, and approximately 30% to 40% of patients with advanced lung cancer develop bone metastasis, which explains the high mortality rates and poor quality of life.^4^, ^5^ Osteolytic metastasis is associated with enhanced osteoclast activity.^6^ Receptor activator of NF‐kB ligand (RANKL) signaling is essential for the terminal differentiation of monocytes/macrophages into osteoclasts.^7^ Increased RANKL expression in the tumoral bone environment can increase osteoclast differentiation and bone resorption activity, resulting in bone metastasis.^8^ In our previous study, a group of organ‐specific metastatic cell lines which only metastasize to the spinal column, lung, brain, and mediastinal lymph node were successfully established from the parent lung cancer cell line L9981‐Luc.^9^ The four cell lines were: L9981‐BoM, L9981‐LuM, L9981‐BrM and L9981‐LnM, respectively.^9^ Compared to the parent cell line (L9981‐Luc), the morphology and biological behavior of the four organ‐specific metastatic cells changed significantly.^9^ Given this, it will be helpful to provide reliable cell model for further studying the molecular mechanisms and signal regulation of organ‐specific metastasis in lung cancer.

MicroRNAs (miRNAs) are an abundant class of small, non‐coding RNAs, approximately 19–25 nucleotides long.^10^ They modulate the expression of target genes by interacting with the 3' untranslated regions (3'‐UTRs) of target mRNA and play an essential role in the biological and pathological processes of various diseases.^11^, ^12^ Many studies have also indicated that microRNAs can modulate tumor initiation and progression and function in tumor cell invasion and metastasis.^13^, ^14^, ^15^, ^16^ Studies have shown that miR‐660‐5p regulates the malignancy of breast cancer cells by suppressing the expression of TFCP2, and is a novel therapeutic target for clinical treatment and a potential prognostic indicator.^17^, ^18^ Moreover, miR‐660‐5p acts as a tumor suppressor in renal cell carcinoma and may regulate cell migration, proliferation, and apoptosis.^19^ However, the role of miR‐660‐5p in the pathogenesis of lung cancer remains unknown. This study aimed to elucidate the role of miR‐660‐5p in organ‐specific metastasis of lung cancer cells and the molecular mechanism underlying its functions.

The SMARCA5 (SWI/SNF‐related, matrix‐associated, actin‐dependent regulator of chromatin, subfamily a, member 5) belongs to the ISWI family of chromatin remodelers which have helicase and ATPase activities and are thought to regulate transcription of specific genes by altering the chromatin structure around them.^20^, ^21^ The chromosome location that determined for the SMARCA5 gene, 4q31, is within a region where loss of heterozygosity (LOH) is frequently observed in hepatocellular carcinomas.^22^, ^23^ Furthermore, studies indicate that SMARCA5 expression is dysregulated in many human malignant tumors, such as gastric cancer, breast cancer, prostate cancer, and acute leukemia.^24^, ^25^, ^26^, ^27^ Also, SMARCA5 gene is a target of miRNA regulation.^28^, ^29^ However, the association between SMARCA5 expression and the formation, progression, and metastasis of lung cancer cells remains unclear.

The Nm23‐H1 gene was initially identified as a suppressor of tumor metastasis.^30^, ^31^, ^32^ The downregulation and hetero‐deletion of the gene are closely correlated with the high metastatic potential and poor prognosis of lung cancer.^33^, ^34^ Besides, the aberration of the nm23‐H1 gene is often companied by alteration in tumor metastasis‐related genes. Nm23‐H1 serves as an upstream regulatory gene that inhibits lung cancer metastasis by regulating downstream genes.^35^, ^36^

The results of the present study showed that nm23‐H1 expression was negatively correlated with miR‐660‐5p expression in organ‐specific metastatic lung cancer cells. Also, the regulation of miR‐660‐5p through nm23‐H1 was confirmed. Downregulation of miR‐660‐5p inhibited the proliferation, migration, invasion, and bone‐specific metastasis of lung cancer cells. Furthermore, the chromatin remodeler SMARCA5 was identified as one of the direct target genes of miR‐660‐5p. Overexpression of SMARCA5 suppressed lung cancer cell migration and invasion. In addition, miR‐660‐5p activated the RANKL signaling through SMARCA5 suppression. These results demonstrated that miR‐660‐5p could be a potential therapeutic target for lung cancer metastasis.

## Methods

### Cell lines and cell culture

The subcell lines (high‐metastatic L9981 and low‐metastatic NL9980) were isolated from a human lung large cell carcinoma cell line and established.^37^ A group of organ‐specific metastatic cell lines: L9981‐BoM, L9981‐LuM, L9981‐BrM, and L9981‐LnM were established from the parent lung cancer cell line L9981‐Luc.^9^ The cell lines with stable nm23‐H1 gene silencing (ie, NL9980‐99 and A549‐99) were established from NL9980 and A549 cell lines.^38^ The A549 cell line was procured from the American Tissue Culture Collection (ATCC). Cells were cultured in RPMI‐1640 medium (Gibco, Carlsbad, CA, USA) with 10% fetal bovine serum (FBS; Gibco) and 1% penicillin‐streptomycin (Sigma Aldrich, Saint Louis, MO, USA) at 37°C, in a humidified atmosphere of 95% air and 5% CO_2_.

### Quantitative real‐time PCR

Quantitative RT‐PCR was performed to validate the miRNA and mRNA expression levels using SYBR Premix Ex Taq (Takara, Japan). The PCR was performed in triplicate and analyzed using the ABI Prism 7900HT fast real‐time PCR system (Applied Biosystems, Life Technologies, USA). The relative quantification values for each gene were calculated by the 2^−ΔΔCt^ method using U6 or GAPDH as an internal reference. The primer for miR‐660‐5p was a specific miRNA primer (Hsa‐miR‐660‐5p; Ruibobio, Guangzhou, China). The other primers in this study are shown in Table S1.

### Cell transfection

To study miR‐660‐5p gene regulation, 3 × 10^5^ cells were seeded into six‐well plates and cultured for 24 hours at 37°C before transfection. A total of 200 pmol of synthesized miR‐660‐5p mimic or inhibitor (GenePharma, Shanghai, China) were transfected into L9981‐Luc or L9981‐BoM cell lines (once the cells had reached 60%–80% confluence) using Lipofectamine 2000 (Invitrogen, CA, USA) according to the manufacturer's instructions. The same quantities of mimic or inhibitor were used as negative controls (NCs; GenePharma, Shanghai, China). Subsequently, transfection efficiency was verified by RT‐qPCR.

### Cell migration assays

To conduct a wound‐healing migration assay, the cells were seeded onto 35 mm dishes coated with fibronectin. Once the cells had reached 100% confluence, a scratch (approximately 500 μm long) was created on the confluent monolayer using a sterile p200 pipette tip. The cell debris was then removed by replacing the medium with fresh serum‐free medium. During the subsequent 48 hours culture of the cells, the width of the wound was measured at 0 hours, 24 hours, and 48 hours time points. Three to four different locations were visualized and photographed under a phase‐contrast inverted microscope. Images were taken in 40x field of vision.

### Cell invasion assays

A Matrigel transwell chamber (BD), filled with the serum‐free medium, was used for the invasion assay. A total of 1 × 10^4^ cells were loaded into the upper chamber. Medium containing 20% FBS was used as the chemoattractant in the lower chamber. After 24 hours of incubation at 37°C, the cells that had adhered to the lower membrane were fixed, stained, and counted using a microscope and a counting chamber (Olympus, Tokyo, Japan). Photos were taken in 100x field of vision.

### Cell proliferation assay

Cell Counting Kit‐8 (CCK‐8) assay was used to analyze cell proliferation. Cells were seeded in 96 well plated at 5.0 × 10^3^ cells/mL and cultured for 24 hours, 48 hours, and 72 hours durations. At the end of each time point, the cells were stained with 10 μL of CCK‐8 solution (Dojindo, Tokyo, Japan) then incubated for 3 hours at 37°C. Each absorbance was measured at 450 nm (A450) using a spectrophotometer. Each sample was run in triplicate.

### Generation of stable miR‐660‐5p and nm23‐H1 overexpressing cells

Experiments were performed using lentiviral vector (GenePharma, Shanghai, China) to obtain stable miR‐660‐5p and nm23‐H1 overexpressing cells. Lung cancer cells were seeded at 5 × 10^4^ cell density in each 24 well plate and infected with miR‐660‐5p/nm23‐H1/control lentiviral vector at multiplicity of infection of 10 (10 infectious units for each target cells). After 72 hours of infection, cells were selected with Puromycin, and mRNA overexpression was quantified on day 10 or 30 after infection. A group of stable miR‐660‐5p or nm23‐H1 overexpressing cells labelled L9981‐Luc‐miR‐660‐5p, L9981‐BoM‐nm23‐H1, and L9981‐BoM‐nm23‐H1‐miR‐660‐5p were established.

### In vivo assay

For in vivo metastasis assays, the stable cell lines L9981‐Luc‐miR‐660‐5p, L9981‐BoM‐nm23‐H1, and L9981‐BoM‐nm23‐H1‐miR‐660‐5p and control cell lines L9981‐luc‐Lv‐NC and L9981‐BoM‐Lv‐NC were collected and suspended in 0.2 mL PBS for each mouse (five mice in each group, 6–8 weeks old), and the cells were injected into the left side of the posterior flank of the nude mice. A total of 30 minutes after cell injection, luciferase substrate was injected at a dose of 150 mg/kg, and live images of the mice were obtained using an IVIS200 (Xenogene, USA). These initial data were regarded as those for Day 0. Luciferase activity was measured every seven days using the same protocol. Tumor growth was measured periodically. Image‐Pro software (Media Cybernetics, Bethesda, MD, USA) was used to calculate the fluorescence intensity. Bone metastasis rate was evaluated according to the number of spinal column metastases in each group.

### Dual‐luciferase reporter assay

The full‐length 3'untranslated region (3'UTR) of SMARCA5 amplified from human genomic DNA was cloned into the downstream of the firefly luciferase coding region of pMIR‐GLOTM Luciferase vector (Promega, USA). The recombined vector was denoted as pMIR‐SMARCA5. Mutations of miR‐660‐5p binding sites were introduced by site‐directed mutagenesis, and the resulting vector was denoted as pMIR‐SMARCA5‐Mut. Cells were seeded into 24 well plates and cotransfected with 200 ng of pMIR‐SMARCA or pMIR‐SMARCA‐Mut vectors and 100 ng of miR‐660‐5p mimic or mimic control. The pRL‐TK plasmid (Promega, Madison, WI) was used for internal normalization. After 48 hours, cells were lysed using the lysis buffer (Promega). Luciferase reporter gene assay was conducted using the Dual‐Luciferase Reporter Assay System (Promega) according to the manufacturer's instructions. All experiments were performed at least three times.

### Western blotting

Cells were transfected with either miR‐660‐5p, pcDNA3.1‐SMARCA5, or small interfering RNA targeting SMARCA5 (si‐SMARCA5; GenePharma, Shanghai, China). Total cellular protein extracted using RIPA buffer (Beyotime, China) were resolved on 10% gradient SDS‐PAGE (sodium dodecyl sulphate‐ polyacrylamide gel) then transferred onto nitrocellulose (NC) membranes. Membranes were blocked for one hour in 5% skim milk in TBST and incubated with primary antibody overnight at 4°C, followed by the incubation with appropriate horseradish peroxidase (HRP) ‐conjugated secondary antibody at optimized concentration. The primary antibodies used in this study were as follows: anti‐nm23‐H1 antibody (1:500, CST), anti‐SMARCA5 antibody (1:2000, CST), anti‐RANKL antibody (1:1000, CST) and anti‐β‐actin antibody (1:3000, CST). The densitometry of western blot results was measured using ImageJ software.

### Statistical analysis

The data are presented as mean ± standard deviation (SD). Student's *t*‐test was used to determine the significant differences between control and treatment groups. All statistical analyses were performed using SPSS15.0 software, and *P* < 0.05 was considered to be a statistically significant difference.

## Results

### Downregulation of nm23‐H1 expression in lung cancer cells results in upregulation of miR‐660‐5p expression

**Figure 1:**
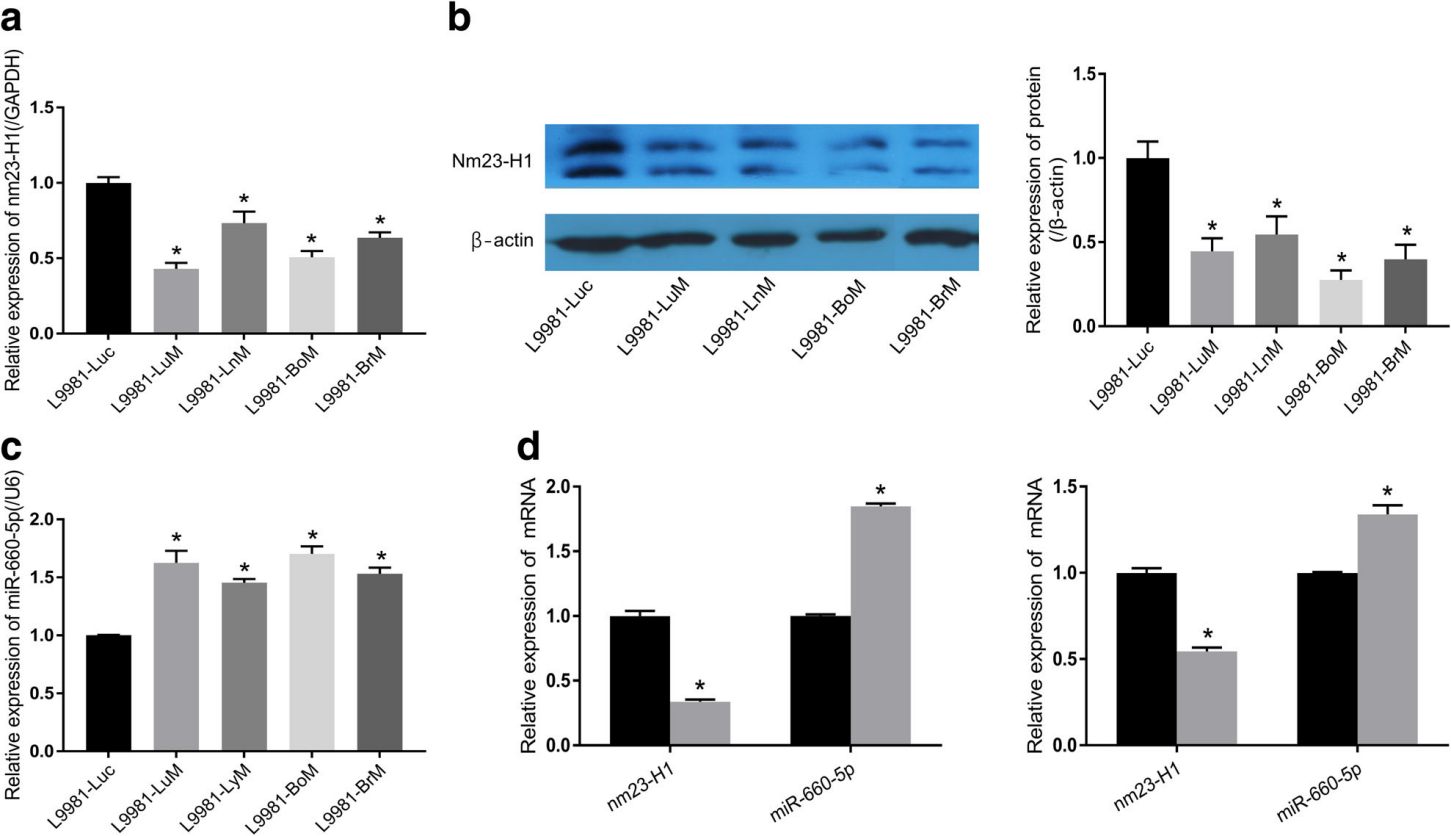
Crosstalk between nm23‐H1 and miR‐660‐5p gene expressions in lung cancer cells. (**a**) The relative mRNA levels of nm23‐H1 as detected by RT‐PCR analysis in normal L9981‐Luc and organ‐specific metastasis lung cancer cells. (**b**) The Nm23‐H1 protein expression as determined by western blot in normal L9981‐Luc and organ‐specific metastatic cells. (**c**) The relative mRNA levels of miR‐660‐5p as detected by RT‐PCR in L9981‐Luc and organ‐specific metastatic cells. (**d**) The relative mRNA levels of nm23‐H1 and miR‐660‐5p in lung cancer cells and cells with stable nm23‐H1 gene silencing (*P < 0.05, *t*‐test). (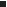) NL9980 and (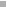) NL9980‐99. (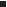) A549 and (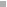) A549‐99.

In the present study, qRT‐PCR and western blot assays were used to evaluate the expression of nm23‐H1 in normal L9981‐Luc and organ‐specific metastatic cells, including L9981‐LuM, L9981‐LyM, L9981‐BoM, and L9981‐BrM. The results showed that nm23‐H1 downregulated significantly both mRNA and protein level in all the organ‐specific metastatic cell lines compared with the normal L9981‐luc cell line (Fig 1a,b). Also, the expression of miR‐660‐5p was analyzed in the same cell lines, and the results showed that miR‐660‐5p was upregulated in all the cell lines with organ‐specific metastasis potential (Fig 1c). Furthermore, the increased expression of miR‐660‐5p in L9980‐99 and A549‐99 cells with low expression of nm23‐H1 was validated (Fig 1d). These findings indicate that nm23‐H1 negatively regulates miRNA‐660‐5p, and the enhanced expression of miR‐660‐5p is associated with organ‐specific metastasis of lung cancer cells.

### Downregulation of miR‐660‐5p inhibits growth, migration, and invasion of lung cancer cells with bone‐specific metastatic potential in vitro

**Figure 2:**
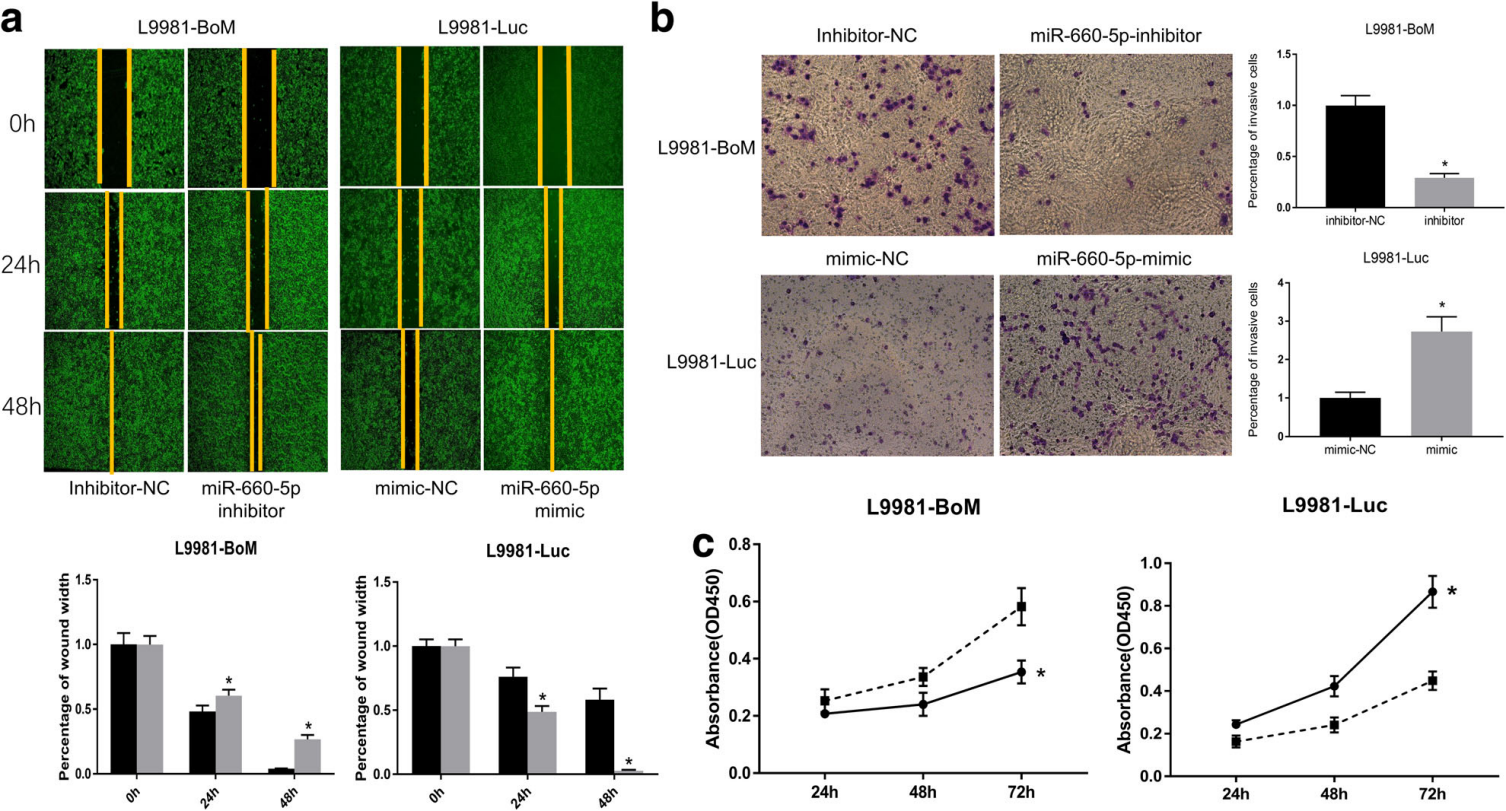
Effect of miR‐660‐5p suppression on the growth, migration, and invasion of bone‐specific metastatic cells in vitro. (**a**) The wound‐healing assay results showing the migration ability of L9981‐BoM and L9981‐Luc cells. Cells were transfected with miR‐660‐5p inhibitor or inhibitor control and miR‐660‐5p mimic or mimic control and photos were taken in 40x field of vision. L9981‐BoM (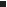) inhibitor‐NC and (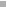) inhibitor. L9981‐Luc (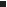) mimic‐NC and (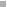) mimic. (**b**) Boyden chamber assay results showing the invasion ability of L9981‐BoM and L9981‐Luc cells. The results were from three independent experiments. The migratory cell number in each group was normalized to the control. Cells were transfected with miR‐660‐5p inhibitor or inhibitor control, and miR‐660‐5p mimic or mimic control. Images were taken in 100x field of vision. L9981‐BoM (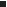) inhibitor‐NC and (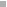) inhibitor. L9981‐Luc (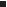) mimic‐NC and (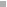) mimic. (**c**) The CCK‐8 assay results showing the proliferation ability of L9981‐BoM and L9981‐Luc cells. Cells were transfected with miR‐660‐5p inhibitor or inhibitor control and miR‐660‐5p mimic or mimics control (*P < 0.05, *t*‐test). L9981‐BoM (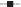) inhibitor‐NC and (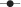) inhibitor. L9981‐Luc (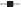) mimic‐NC and (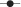) mimic.

Next, the functional significance of miR‐660‐5p in lung cancer cells was evaluated. Briefly, L9981‐BoM cells with high MiR‐660‐5p expression were transfected with miR‐660‐5p inhibitor or inhibitor control, whereas L9981‐Luc cells with low miR‐660‐5p expression were transfected with miR‐660‐5p mimics or NCs. A wound‐healing assay was used to examine cell migration ability. The downregulation of miR‐660‐5p in L9981‐BoM significantly inhibited cell migration compared to the control group (Fig 2a). Furthermore, the Boyden chamber assay was conducted to investigate the effect of miR‐660‐5p on cell invasion. The invasion ability of L9981‐BoM cells transfected with miR‐660‐5p inhibitor was lower than that of the control group (Fig 2b). Moreover, a CCK‐8 assay was performed to investigate the effect of miR‐660‐5p on cell proliferation. The proliferation ability of L9981‐BoM cells transfected with miR‐660‐5p inhibitor was lower than that of the control group (Fig 2c). However, the normal L9981‐Luc cells showed an increased migration, invasion and proliferation after treatment with miR‐660‐5p mimics (Fig 2a–c). Collectively, these results suggest that miR‐660‐5p can be an oncogene, and thus inhibiting miR‐660‐5p can suppress the growth, migration, and invasion of bone‐specific metastatic cells in vitro.

### MiR‐660‐5p promotes lung cancer progression and bone‐specific metastasis and reverse the antitumor effect of nm23‐H1 in vivo

**Figure 3:**
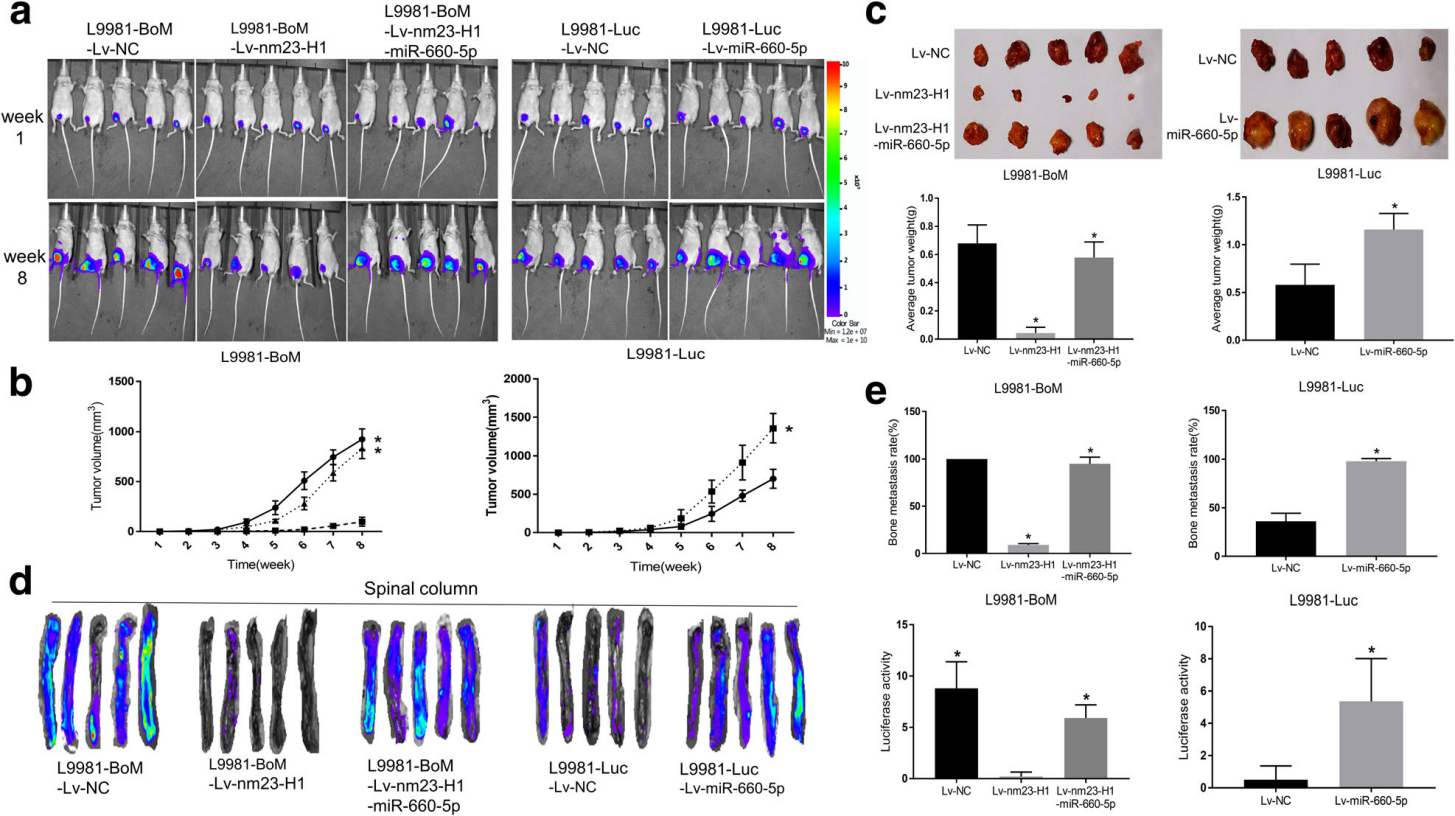
MiR‐660‐5p promotes lung cancer progression and bone metastasis and reverses the anti‐tumor effect of nm23‐H1 in vivo. The miR‐660‐5p or nm23‐H1 overexpressed cells L9981‐Luc‐miR‐660‐5p, L9981‐BoM ‐Lv‐nm23‐H1, L9981‐BoM ‐Lv‐nm23‐H1‐miR‐660‐5p and negative control cells L9981‐Luc‐Lv‐NC, L9981‐BoM‐Lv‐NC were injected into the nude mice (5/group). (**a**) Representative images of tumors (durations are indicated). The pseudo‐color scale bars represent the intensity of light emission with different colors. (**b**) Tumor growth curve (data are mean ± SD). L9981‐BoM (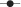) Lv‐NC, (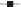) Lv‐nm23‐H1 and (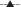) Lv‐nm23‐H1‐miR‐660‐5p. L9981‐Luc (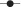) Lv‐NC and (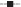) Lv‐miR‐660‐5p. (**c**) Images of tumors obtained from different groups of nude mice (up) and a comparison of the average weight of implanted tumors (down). L9981‐BoM (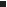) Lv‐NC, (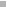) Lv‐nm23‐H1 and (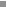) Lv‐nm23‐H1‐miR‐660‐5p. L9981‐Luc (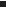) Lv‐NC and (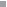) Lv‐miR‐660‐5p. (**d**) Spinal column metastases luciferase imaging of tumor‐bearing mice in vitro. (**e**) Comparison of bone metastasis rate (up) and the luciferase value (down) of each group based on spinal metastasis (the intensity was measured on week 8 and data are presented as mean ± SD) (*P < 0.05, *t*‐test). L9981‐BoM (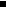) Lv‐NC, (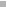) Lv‐nm23‐H1 and (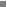) Lv‐nm23‐H1‐miR‐660‐5p. L9981‐Luc (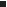) Lv‐NC and (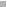) Lv‐miR‐660‐5p. L9981‐BoM (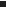) Lv‐NC, (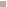) Lv‐nm23‐H1 and (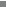) Lv‐nm23‐H1‐miR‐660‐5p. L9981‐Luc (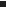) Lv‐NC and (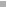) Lv‐miR‐660‐5p.

To further determine the roles of miR‐660‐5p, L9981‐Luc cells with stable overexpression of miR‐660‐5p were generated and injected subcutaneously into nude mice. Subsequently, tumor growth was closely monitored for eight weeks. The fluorescence value of mice in the miR‐660‐5p overexpressed group was significantly higher than the negative control group (Fig 3a). The tumor size and weight were also increased significantly in the miR‐660‐5p overexpressed group (Fig 3b,c). Furthermore, spinal column metastases fluorescence value of tumor‐bearing mice in vitro was used to investigate the effect of miR‐660‐5p on bone metastasis (Fig 3d). The bone metastasis rate and fluorescence value in the miR‐660‐5p overexpressed group were significantly higher than those of the control group (Fig 3e).

This study also established L9981‐BoM ‐Lv‐nm23‐H1 (nm23‐H1) cell lines with high nm23‐H1 expression and L9981‐BoM ‐Lv‐nm23‐H1‐miR‐660‐5p cell lines with high miR‐660‐5p expression and another L9981‐BoM‐Lv‐con cell line as a negative control (NC). The cells were injected subcutaneously to the nude mice, and the fluorescence intensity was measured every week until the eighth week. The nm23‐H1 overexpressed group showed a significant decrease in luciferase activity, tumor size, and weight compared with the negative control group (Fig 3a–c). Also, overexpression of nm23‐H1 significantly decreased the number of bone metastasis and metastases fluorescence value compared with the negative control group (Fig 3d,e). In addition, compared with the nm23‐H1 overexpressed group, the tumor size and weight, bone metastasis rate, and luciferase activity were increased in the re‐induced miR‐660‐5p group, but the increase was not significantly higher than the negative control group (Fig 3a–e).

### MiR‐660‐5p directly inhibits SMARCA5 expression via targeting its 3'UTR and induces RANKL signaling in lung cancer cells

**Figure 4:**
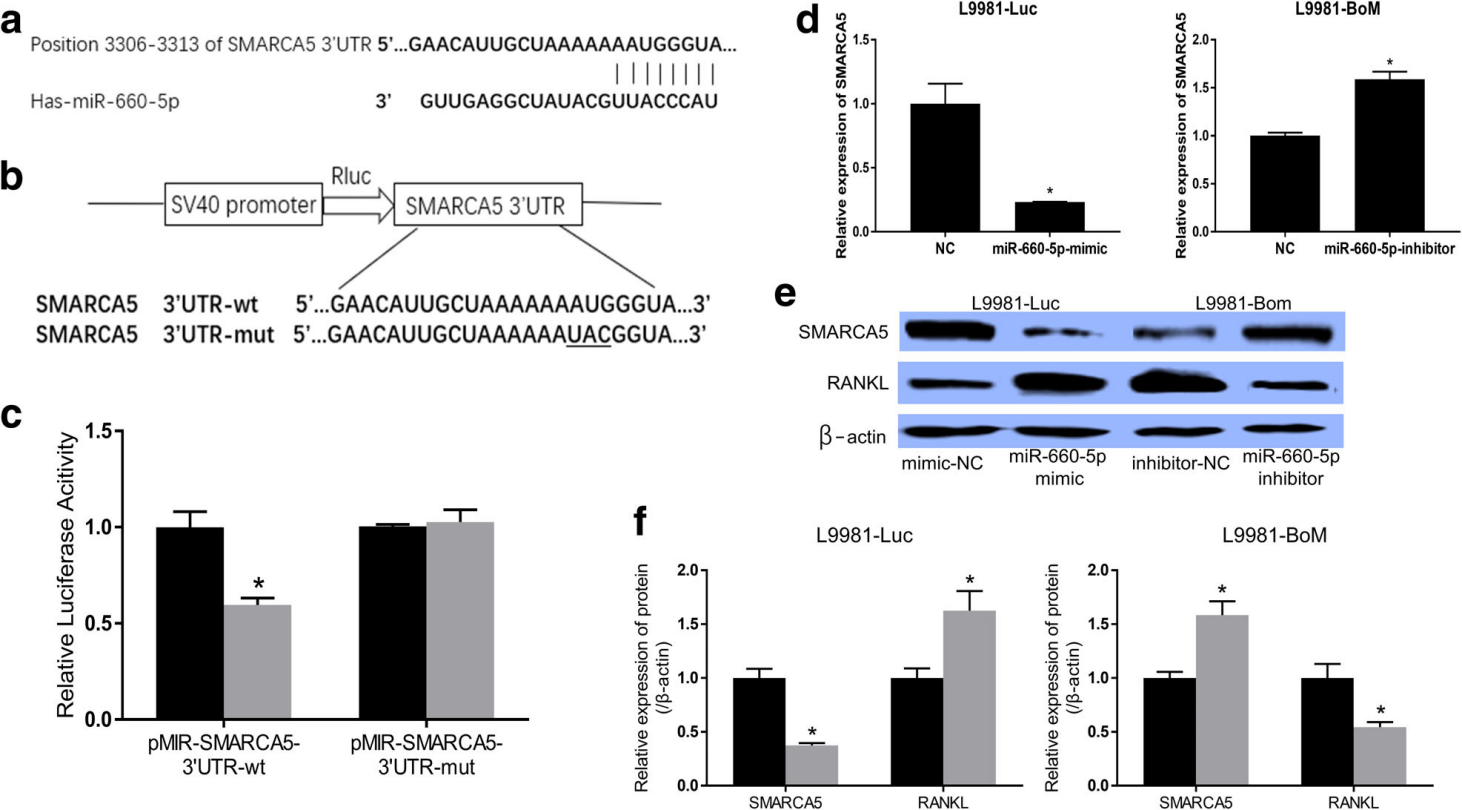
MiR‐660‐5p inhibits the expression of SMARCA5 through its 3'UTR. (**a**) The miR‐660‐5p binding site, as predicted in the 3'UTR of SMARCA5 mRNA. (**b**) Mutation generated at the seed region of SMARCA5 3'UTR (indicated by the underlined bases). A 3'UTR fragment of SMARCA5 mRNA containing wild‐type or mutant of the miR‐660‐5p binding sequence was cloned into the downstream of the luciferase gene in the pMIR vector. (**c**) L9981‐Luc cells were transfected with pMIR reporter vectors containing either wild‐type or mutant SMARCA5 3'UTR (indicated as pMIR‐SMARCA5‐3'UTR‐wt and pMIR‐SMARCA5‐3'UTR‐mut) with either miR‐660‐5p mimics (indicated as miR‐660‐5p) or miR‐660‐5p mimics control (indicated as NC). Bar graphs showing luciferase activity 48 hours after transfection. (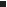) NC and (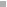) miR‐660‐5p. (**d**–**f**) The mRNA and protein expression levels of SMARCA5 and RANKL genes as determined by RT‐PCR and western blot assays in L9981‐Luc or L9981‐BoM cell transfected with miR‐660‐5p mimic, control miRNA mimic, miR‐660‐5p inhibitor, or control miRNA inhibitor. (**e**, **f**) Here the relative gray values of each band (normalized to β‐actin) are shown. Protein bands from three independent western blot assays were quantified using Quantity One software (Bio‐Rad, USA). Data are presented as mean ± SD (*P < 0.05, *t*‐test). L9981‐Luc (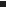) mimic‐NC and (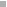) miR‐660‐5p mimic. L9981‐BoM (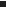) inhibitor‐NC and (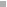) inhibitor.

The phenotypic results mentioned above indicate that the growth and migration of the human lung cancer cells with bone‐specific metastasis potential were both reduced by the inhibition of miR‐660‐5p. Moreover, TARGETSCAN, MIRDB, and MICRORNA.ORG were used to predict the targets of miR‐660‐5p. Notably, the results of the present study suggested that SMARCA5 was indeed a potential target of miR‐660‐5p (Fig 4). The 3'‐UTR of SMARCA5 mRNA contained a site that complemented the seed region of miR‐660‐5p (Fig 4a). The human SMARCA5 3'‐UTR fragments with wild‐type or mutant miR‐660‐5p‐binding sequences were cloned into pMIR reporter vector (Fig 4b). The luciferase activity of pMIR‐SMARCA5‐3'UTR‐wt construct was significantly decreased (because of the overexpression of miR‐660‐5p in L9981 cells), whereas its mutant counterpart was not (Fig 4c). Moreover, the expression levels of SMARCA5 were determined in L9981‐Luc and L9981‐BoM cells transfected with miR‐660‐5p mimics or inhibitors and miR‐NC. The mRNA and protein levels of SMARCA5 in L9981 cells were dramatically reduced after transfection with miR‐660‐5p mimics. Also, the SMARCA5 mRNA and protein levels were higher in the L9981‐BoM cells than in the miRNA control as a result of miR‐660‐5p suppression (Fig 4d–f). Collectively, these results indicate that SMARCA5 is a direct target of miR‐660‐5p.

RANKL plays a critical role in the development of osteolytic metastasis in the bones.^39^ It is highly expressed in the osteolytic lesions associated with malignant tumors.^40^ Moreover, by blocking the RANKL signaling, osteolytic lesions have been successfully inhibited in several types of cancer, including multiple myeloma and prostate cancer.^41^, ^42^, ^43^ Given this, the present study evaluated the effect of miR‐660‐5p on RANKL in lung cancer cells using western blot assay. The expression of RANKL was positively correlated with miR‐660‐5p expression in L9981‐Luc or L9981‐BoM cells (4E). Collectively, these results show that miR‐660‐5p directly inhibits SMARCA5 expression by targeting its 3'UTR and also induces RANKL signaling of lung cancer cells.

### SMARCA5 expression is inversely correlated with miR‐660‐5p mediated bone‐specific metastatic lung cancer cell migration, invasion, and RANKL signaling

**Figure 5:**
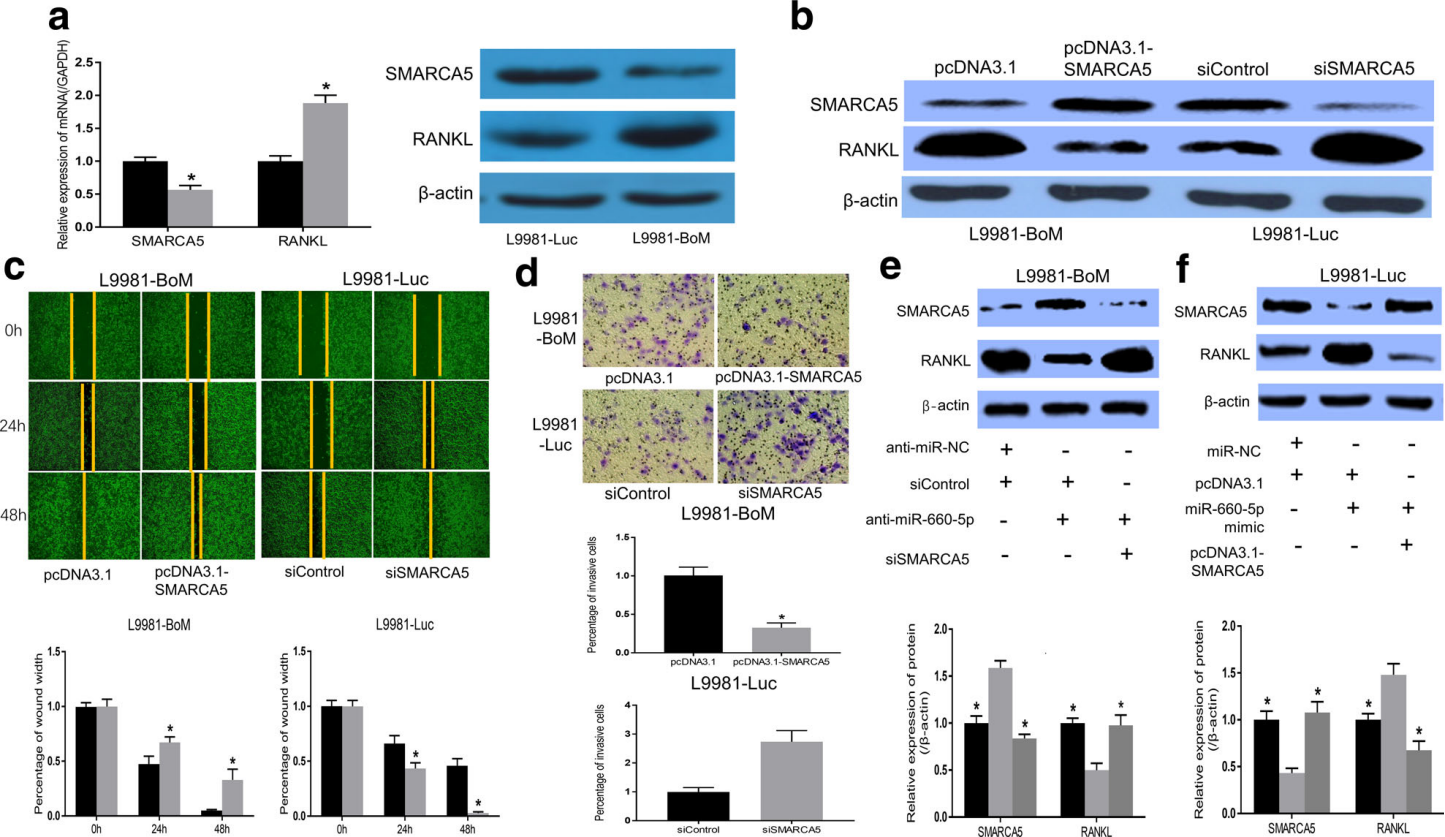
Effect of inverse correlation between SMARCA5 and miR‐660‐5p expressions on bone‐specific metastatic lung cancer cell migration, invasion, and RANKL signaling. (**a**) Results of RT‐PCR and western blot assays showing SMARCA5 and RANKL expressions as examined in L9981‐Luc and L9981‐BoM cells. (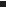) L9981‐Luc and (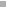) L9981‐BoM. (**b**) SMARCA5 and RANKL expression as revealed by western blot assay after transfecting pcDNA3.1‐SMARCA5 in L9981‐BoM or siSMARCA5 in L9981‐Luc to overexpression or knockdown of SMARCA5. (**c**) The cell migration ability, as detected by wound healing assay in L9981‐BoM cells, transfected with pcDNA3.1 or pcDNA3.1‐SMARCA5 vectors and L9981‐Luc transfected with siSMARCA5 or siControl. Images were taken in 40x field of vision. L9981‐BoM (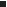) pcDNA3.1 and (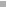) pcDNA3.1‐SMARCA5. L9981‐Luc (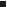) siControl and (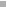) siSMARCA5. (**d**) The effect of SMARCA5 on the cell invasion as assessed by the Boyden chamber assay after overexpression or knockdown of SMARCA5 in L9981‐BoM or L9981‐Luc cells, respectively. Images were taken in 100x field of vision. L9981‐BoM (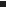) pcDNA3.1 and (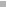) pcDNA3.1‐SMARCA5. L9981‐Luc (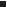) siControl and (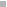) siSMARCA5. (**e**, **f**) The SMARCA5 and RANKL protein expression levels as determined by western blot (*P < 0.05, *t*‐test). (**e**) (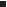) anti‐miR‐NC+siControl, (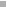) anti‐miR‐660‐5p+siControl and (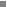) anti‐miR‐660‐5p+siSMARCA5. (**f**) (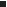) miR‐NC‐pcDNA3.1, (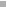) miR‐660‐5p+pcDNA3.1 and (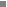) miR‐660‐5p+pcDNA3.1‐SMARCA5.

The expression of SMARCA5 and RANKL in normal L9981‐Luc and bone‐specific metastatic L9981‐BoM cells was also examined in this study. Both the mRNA and protein levels of SMARCA5 in L9981‐BoM cells were significantly low. Also, RANKL expression was higher in bone‐specific metastatic L9981‐BoM cells than in the normal L9981‐Luc cells (Fig 5a). A significant reverse correlation between SMARCA5 and RANKL (Fig 5b) was observed after transfecting pcDNA3.1‐SMARCA5 in L9981‐BoM or siSMARCA5 in L9981‐Luc cells for overexpression or knockdown of SMARCA5, respectively. Furthermore, we investigated whether SMARCA5 contributed to the migration and invasion of bone‐specific metastatic lung cancer cells. Ectopic expression of SMARCA5 in L9981‐BoM cells led to a significant decrease in cell migration and invasion (Fig 5c,d); however, silencing SMARCA5 by siRNAs in L9981 cells resulted in increased migration and invasion of the cells (Fig 5c,d). Therefore, SMARCA5 was negatively correlated with bone‐specific metastatic cell migration and invasion.

In the present study, miR‐660‐5p regulated the expression of SMARCA5 and induced RANKL signaling. Also, there was a significant negative correlation between SMARCA5 and RANKL expressions. Given this, we investigated whether the role of miR‐660‐5p on RANKL signaling was dependent on SMARCA5. The expression level of RANKL was significantly reduced or increased in L9981‐BoM, and L9981‐Luc cells transfected with miR‐660‐5p inhibitor or mimic, respectively (Fig 5e,f). After SMARCA5 silencing, RANKL inhibition induced through miR‐660‐5p was reversed in L9981‐BoM cells (Fig 5e). Thus, SMARCA5 could be involved in the regulation of miR‐660‐5p on RANKL signaling in L9981‐BoM cells. Also, the overexpression of SMARCA5 could reverse the increased RANKL expression induced by miR‐660‐5p in L9981‐Luc cells (Fig 5f). Collectively, these results suggest that SMARCA5 is a functional target of miR‐660‐5p, which is inversely correlated with miR‐660‐5p mediated bone‐specific metastatic lung cancer cells migration, invasion, and RANKL signaling.

## Discussion

Tumor cells tend to spread (metastasize) to adjacent and distant tissues and organs which poses a considerable challenge to cancer treatment and management. Metastasis is the leading cause of death in cancer patients.^44^ Lung cancer metastasis has organ‐specific characteristics. For studying the mechanisms and signal regulation of organ‐specific metastasis of lung cancer, our laboratory screened and established an organ‐specific metastatic lung cancer cell model from the parent lung cancer cell line L9981‐Luc, including L9981‐BrM, L9981‐LuM, L9981‐BoM, L9981‐LnM, which are only able metastasized to brain, lung, spinal column and mediastinal lymph nodes.^9^ The nm23‐H1 gene is a well‐known suppressor of tumor metastasis. In this study, nm23‐H1 was significantly downregulated in all four organ‐specific metastasis cell lines, which suggested that organ‐specific metastasis could occur as a result of nm23‐H1 suppression. The downregulation of nm23‐H1 might influence the expression of some genes downstream.

Several lines of evidence have indicated that miRNAs play a crucial role in tumor formation and progression.^13^, ^14^, ^15^, ^16^ Previous studies have revealed that miR‐660‐5p is dysregulated in several human malignancies. For example, miR‐660‐5p is upregulated in breast cancer, and it may be a potential oncogene that plays a vital role in the development and metastasis of breast cancer.^17^ Another study reported that miR‐660‐5p expression was significantly downregulated in renal cell carcinoma and could act as a tumor suppressor to regulate cell migration, proliferation, and apoptosis.^19^ Also, dysregulated miR‐660‐5p expression has been reported in Hodgkin's lymphoma^45^ and multiple myeloma.^46^ In the present study, miR‐660‐5p was significantly upregulated in all four organ‐specific metastasis cell lines with lower nm23‐H1 expression than normal L9981‐Luc. Thus, we speculated that negative regulation of miR‐660‐5p by nm23‐H1 might be involved in the organ‐specific metastasis of lung cancer cells. However, the role of miR‐660‐5p in lung cancer cell migration and invasion remains unclear.

Herein, the role of miR‐660‐5p in lung cancer cell progression was investigated. The downregulation of miR‐660‐5p in bone‐specific metastatic lung cancer cell L9981‐BoM significantly inhibited cell proliferation, migration, and invasion in vitro, whereas the upregulation of miR‐660‐5p in L9981‐Luc significantly promoted lung cancer tumorigenesis and bone‐specific metastasis in vitro and in vivo. Moreover, the reintroduction of miR‐660‐5p reversed nm23‐H1 mediated antitumor and antibone‐specific metastatic effect in L9981‐BoM in vivo. These results suggested that miR‐660‐5p could serve as a downstream gene of nm23‐H1, and could be a novel potential oncogene that plays a vital role in the development and bone‐specific metastasis of lung cancer.

The biological function of miRNAs is to regulate target genes by directly inactivating mRNA or inhibiting protein synthesis.^11^, ^12^ In the current study, it was revealed that SMARCA5 has a binding site for miR‐660‐5p within its 3'‐UTR region, as earlier predicted. Furthermore, SMARCA5 was characterized as a functional target of miR‐660‐5p by luciferase reporter gene assays, RT‐PCR, and western blot analysis. The imitation switch (ISWI) nuclear ATPase SMARCA5 (Snf2h) is one of the most conserved chromatin remodeling factors which are associated with various developmental disorders and cancer.^21^, ^22^ A study reported that SMARCA5 expression was higher in gastric cancer samples than in normal mucosa^24^ and overexpression of SMARCA5 correlated with cell proliferation and migration in breast cancer.^25^ In the present study, SMARCA5 expression was inversely correlated with miR‐660‐5p expression. Overexpression of SMARCA5 suppressed the L9981‐BoM cell invasion and migration, indicating that SMARCA5 could be involved in the regulation of tumor progression in bone‐specific metastatic lung cancer cells.

Receptor activator of NF‐kB ligand (RANKL) signaling is essential for osteoclastogenesis and bone resorption that contribute to the underlying pathogenesis of tumor cell metastasis to the bone.^7^, ^8^ The RANKL protein expression in cancer cells plays a pivotal role in bone metastasis. Xianbo *et al*. suggested that the movement of lung cancer cells from primary sites to bone metastatic sites could depend on the level of RANKL expression. In their study, the RANKL expression was increased at both transcript and protein levels in the most metastatic cell line.^47^ Moreover, osteolytic metastasis has been successfully inhibited in several types of cancer, including multiple myeloma and prostate cancer through the suppression of RANKL signaling.^41^, ^43^ This study also found similar results that showed that RANKL expression was upregulated in bone‐specific metastasis L9981‐BoM cells compared to L9981‐Luc cells. Also, it was observed that RANKL expression was influenced by miR‐660‐5p expression. The RANKL expression induced by miR‐660‐5p was reversed as a result of SMARCA5 knockdown, indicating that miR‐660‐5p could play a role in bone‐specific metastasis of lung cancer cells by modulating RANKL signaling through targeting SMARCA5. However, more detailed mechanisms involving other relevant downstream factors in the process should be investigated further.

In conclusion, the results of this study show that nm23‐H1 negatively regulates miR‐660‐5p (as a downstream gene). Moreover, miR‐660‐5p influences the proliferation, migration, invasion, and bone‐specific metastasis of human lung cancer cells through the miR‐660‐5p/SMARCA5/RANKL signaling pathways. These findings provide new insight into the mechanism underlying the development of human lung cancer, particularly bone‐specific metastasis. MiR‐660‐5p and its downstream targets may be potential therapeutic targets for the treatment and prognosis of human lung cancer.

## Disclosure

The authors report no conflicts of interest in this work.

## Supporting information


**Table S1** Sequences of components used in this study.
