# Supplementary material for: Nm23‐H1 inhibits lung cancer bone‐specific metastasis by upregulating miR‐660‐5p targeted SMARCA5
Source: Thorac Cancer. 2020 Feb 5;11(3):640–50. doi: 10.1111/1759-7714.13308 (PMC7049508; doi:10.1111/1759-7714.13308)
Supplement: Supplementary file 1 — Table S1 Sequences of components used in this study. [file TCA-11-640-s001.docx]

**Table S1**

**Sequences of components used in this study.**

| Component | Sequence(5’-3’) |
| --- | --- |
| Primers for qRT-PCR,  nm23-H1  SMARCA5  RANKL  U6  GAPDH  Primers for 3’UTR Cloning  SMARCA5 3’UTR-wt  SMARCA5 3’UTR-mut  Transfection  has-miR-660-5p inhibitor  inhibitor NC  hsa-miR-660-5p mimics  mimics NC  si-SMARCA5  si-NC | F: GCAGCCGGAGTTCAAACCTA  R: TGCACACCAGGCTGACTTAG  F: TGCAAACTGACCGGGCAAATA  R: TCGCCAACGGATAGTAAGTTCT  F: CAACATATCGTTGGATCACAGCA  R: GACAGACTCACTTTATGGGAACC  F: CTCGCTTCGGCAGCACA  R: ACGCTTCACGAATTTGCGT  F: TCCAAAATCAAGTGGGGCGA  R: AAATGAGCCCCAGCCTTCTC  F: AGTAGATCTTCAGGCTATGGACC  R: ACTTCTTCTGGAGTTTTGCCTTC  F: TATTTCAGGGAAGCTCTTCGTGTTAGTGA  AATGTTCAG  R: GTGGAGGAAAGAACTGGAAATCCTGAA  CATTTCACT  CAACUCCGAUAUGCAAUGGGUA  CAGUACUUUUGUGUAGUACAA  Sense: UACCCAUUGCAUAUCGGAGUUG  Antisense: ACUCCGAUAUGCAAUGGGUAUU  Sense: UUCUCCGAACGUGUCACGUTT  Antisense: ACGUGACACGUUCGGAGAATT  Sense: CACCGCAACACATGTGTTTGCTTCAT  TCAAGAGATGAAGCAA  Antisense: GATCCAAAAAAGCAACGAGCTC  CAGGACATAGTCTCTTGAAC  Sense: CACCGTATGACAACAGCCTCAAGTT  CAAGAAGACTTGAGGC  Antisense: GATCCAAAAAAGTATGACAACA  GCCTCAAGTCTCTTGAAC |

qRT-PCR, quantitative real-time PCR; nm23-H1, NM23 Nucleoside Diphosphate Kinase 1; SMARCA5, SWI/SNF Related, Matrix Associated, Actin Dependent Regulator of Chromatin, Subfamily A, Member 5; RANKL, Receptor Activator of NF-kB Ligand.
